# MEGA: Maximum-Entropy Genetic Algorithm for Router Nodes Placement in Wireless Mesh Networks

**DOI:** 10.3390/s24206735

**Published:** 2024-10-19

**Authors:** Nurzhan Ussipov, Sayat Akhtanov, Dana Turlykozhayeva, Symbat Temesheva, Almat Akhmetali, Marat Zaidyn, Timur Namazbayev, Aslan Bolysbay, Aigerim Akniyazova, Xiao Tang

**Affiliations:** 1 Faculty of Physics and Technology, Al-Farabi Kazakh National University, Almaty 050040, Kazakhstan; nurzhan.ussipov@kaznu.edu.kz (N.U.); saiyat.ahtanov@kaznu.edu.kz (S.A.); symbat.temesheva@gmail.com (S.T.); akhmetali_almat@kaznu.edu.kz (A.A.); zaidyn_marat@kaznu.edu.kz (M.Z.); timur.namazbayev@gmail.com (T.N.); bolysbay_aslan2@live.kaznu.kz (A.B.); aigerimakniyazova@gmail.com (A.A.); 2 School of Information and Communication Engineering, Xi’an Jiaotong University, Xi’an 710049, China; tangxiao@xjtu.edu.cn

**Keywords:** entropy, genetic algorithm, mesh router nodes placement, network connectivity, user coverage, wireless mesh networks

## Abstract

Over the past decade, wireless mesh networks (WMNs) have seen significant advancements due to their simple deployment, cost-effectiveness, ease of implementation, and reliable service coverage. However, despite these advantages, the placement of nodes in WMNs presents a critical challenge that significantly impacts their performance. This issue is recognized as an NP-hard problem, underscoring the necessity of development optimization algorithms, such as heuristic and metaheuristic approaches. This motivated us to develop the Maximum Entropy Genetic Algorithm (MEGA) to address the issue of mesh router node placement in WMNs. To assess the proposed method, we conducted experiments across various scenarios with different settings, focusing on key metrics such as network connectivity and user coverage. The simulation results showed the comparative performance of MEGA in relation to other prominent algorithms, such as the Coyote Optimization Algorithm (COA), Firefly Algorithm (FA), Genetic Algorithm (GA), and Particle Swarm Optimization (PSO), revealing MEGA’s effectiveness and usability in determining optimal locations for mesh routers.

## 1. Introduction

**Figure 1:**
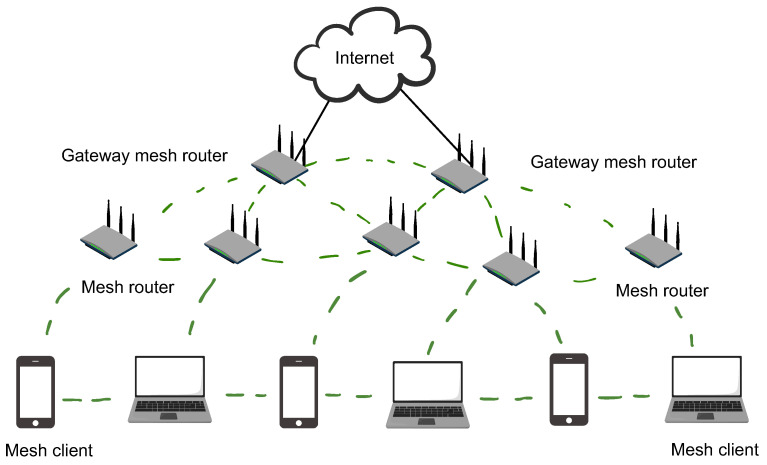
Wireless mesh network architecture.

As an emerging technology, the wireless mesh network (WMN) has gained increasing attention in the communication field during the last decade. This attention has been garnered due to its many advantages, such as quick and easy implementation, dynamic self-organization, self-configuration, extensive network coverage, and cost effectiveness [1,2,3]. WMNs can be deployed in a wide range of applications, e.g., broadband home networking, the education field, healthcare, building automation, disaster management, rescue operations, and military operations [4,5]. A WMN is made up of three different types of nodes: mesh routers (MRs), mesh gateways (MGs), and mesh clients (MCs) as shown in Figure 1. MCs such as laptops, desktops, mobile phones, and other wireless devices connect to the internet via MRs, which transmit traffic to and from MGs. MGs are in turn connected to the internet infrastructure.

Although WMNs have certain desirable characteristics, there are a number of problems preventing their large-scale deployment. One of the critical issues receiving significant attention in the literature is the mesh router nodes placement problem, also known as NP-hard [6,7,8]. The bad position of mesh nodes (MR and/or MG) has a significant impact on WMN performance [9]. Consequently, much interference and congestion occur, leading to substantial packet loss, low throughput, and high delays.

Several papers have presented meta-heuristic algorithms as successful solutions for solving the nodes placement problem in WMNs. Most of them have considered a stationary topology, while others have focused on investigating the dynamic placement of mesh nodes [10,11,12,13,14,15].

To address the stationary variant of the WMN nodes placement problem, Xhafa et al. proposed three algorithms: Simulated Annealing (SA) [16], Hill Climbing (HC) [17], and Tabu Search (TS) [18]. SA effectively avoids local optima, HC offers fast convergence, and TS prevents cycling in the search process. Their performance was evaluated in terms of user coverage and network connectivity.

Sayad et al. introduced the Chemical Reaction Optimization (CRO) algorithm [19], inspired by the intricate interactions between molecules that naturally seek to reach a low and stable energy state. They also developed the Firefly Optimization Algorithm (FA) [20], a robust bio-inspired approach, and conducted a comprehensive evaluation against the established GA. Both CRO and FA demonstrated superior performance in optimizing complex network configurations, effectively handling instances with diverse and varying numbers of mesh clients and routers. These algorithms showcased exceptional adaptability and efficiency, proving their capability to outperform traditional optimization methods in complex scenarios.

Evolutionary algorithms, such as the GA, are also widely used for optimization in this field [21,22,23,24]. Xhafa et al. addressed the issue of mesh router node placement by treating it as a facility location problem and solving it using a GA [21]. Another study introduced an enhanced GA that integrates the Minimum Spanning Tree (MST) to improve cost and coverage outcomes [22]. In [23], an advanced version of GA, named MOGAMESH, was developed to optimize WMN topology by maximizing user coverage and minimizing node degree. Additionally, two variations of GA were explored in [24]: the Non-dominated Sorting Genetic Algorithm-II (NSGA-II) and the Multi-Objective Genetic Algorithm (MOGA), which consider cost, coverage, and reliability as key performance metrics. These studies represent some of the most effective applications of multi-objective algorithms to achieve simultaneous optimization of multiple objectives in this domain [25,26,27].

Recently, Mekhmoukh et al. applied the Coyote Optimization Algorithm (COA) [28] to solve the mesh router node placement problem, outperforming FA, PSO, BA, and other algorithms in terms of user coverage and connectivity. COA is a population-based meta-heuristic that is easy to implement, requiring only two control parameters. It has demonstrated strong performance in solving various placement problems, such as sizing and locating renewable distributed generations, optimal placement of photovoltaic distributed generations, and determining the ideal position of static compensators.

Several methods have been proposed to address the dynamic variant of mesh nodes placement, as discussed in [10,11,12,14]. In [10], an enhanced PSO algorithm incorporating a restriction coefficient into its framework was introduced to tackle this challenge. Similarly, Lin et al. [11] presented an improved bat-inspired algorithm (BA) by integrating a dynamic search scheme into the original BA model. This enhancement was validated through experiments on 10 instances, considering parameters such as coverage and connectivity. The authors in [12] concentrated on the social-aware dynamic placement of router nodes in WMNs. They introduced an enhanced PSO variant termed social-based-PSO, which incorporates a social-supporting vector.

In this work, we propose a new algorithm for the placement of mesh router nodes based on entropy and a genetic algorithm. Notably, the use of an entropy-based approach has not been considered in the methods discussed in this article. In our proposed algorithm, the fitness function is calculated using Shannon’s entropy, aiming to achieve the maximum entropy value [29]. According to this theory, entropy is maximized when the probability distribution of the nodes is uniform. This method of calculating the fitness function through entropy ensures a uniformly distributed placement of mesh router nodes, considering the positions of mesh clients.

Moreover, our approach is inspired by GA, which is known for its robust optimization capabilities. GA imitates the process of natural selection, where the fittest individuals are selected for reproduction to produce the offspring of the next generation. The primary advantages of GA include its ability to efficiently search large and complex spaces, its flexibility in handling various types of objective functions, and its robustness against getting trapped in local optima. We assess the performance of MEGA through numerous simulations with different settings, considering both coverage and connectivity metrics.

The rest of the paper is organized as follows. Section 2 details the formulation of the mesh router nodes placement problem. In Section 3, we introduce a novel entropy and GA inspired algorithm (MEGA), designed to address the mesh router nodes placement problem. Section 4 contains simulation results and comparison with other approximate optimization algorithms. Finally, the conclusion is given in Section 5.

## 2. Mesh Nodes Placement Problem Formulation

In this section, we propose the system model and formulate the problem regarding the placement of mesh router nodes. For better readability, the notations used in this paper are presented in Table 1.

### 2.1. System Model

WMN can be mathematically represented as an undirected graph G=(V,E), where *V* represents the set of network nodes and *E* denotes the links connecting these nodes. The network *G* comprises several disjointed subnetworks. In this work, the WMN includes two types of nodes: mesh clients and mesh routers. Thus, V=M∪C, where:

- *M* is the set of *m* mesh routers, denoted as $M=\{m_{1},m_{2},\ldots ,m_{m}\}$. Each router is equipped with a radio interface having the same coverage radius, denoted as $R_{1}=R_{2}=\ldots =R_{m}$. Two mesh routers, $m_{i}$ and $m_{j}$, can connect only if the distance between them, $d(m_{i},m_{j})$, is less than or equal to twice the coverage radius *R*, i.e., $d(m_{i},m_{j})\leq 2R$.
- *C* is the set of *n* mesh clients, represented as $C=\{c_{1},c_{2},\ldots ,c_{n}\}$. Here, mesh clients are randomly distributed within a two-dimensional rectangular area with dimensions W×H. A mesh client $c_{i}$ is considered covered by a mesh router $m_{j}$ if it falls within the router’s coverage radius, i.e., $d(c_{i},m_{j})\leq R$. Each client can be associated with only one router, typically the nearest one, although it may be within the coverage radius of multiple routers.

### 2.2. Problem Formulation

Depending on the nature of the environments studied (static or dynamic) and the type of deployment spaces (discrete or continuous), various variants of the WMN router nodes placement problem can be identified. In this paper, we focus on the static continuous placement of mesh routers. The primary objective is to determine the optimal positioning of *m* mesh routers within a two-dimensional area with dimensions W×H, taking into account the positions of *n* mesh clients [30,31].

The problem in this article aims to optimize two main objectives:

- User coverage: This refers to the count of users covered by at least one mesh router and can be found according to the formula [28]:
  (1) $$\Psi \left(G\right)=\sum_{i=1}^{n}\left(\max_{j\in \{1,\ldots ,m\}}\sigma_{ij}\right)$$
  where $\sigma_{ij}$ represents the coverage variable, defined as:
  (2) $$\sigma_{ij}=\left\{\begin{array}{cc} 1 & \mathrm{if}\;\mathrm{mesh}\;\mathrm{client}\;c_{i}\;\mathrm{is}\;\mathrm{covered}\;\mathrm{by}\;\mathrm{mesh}\;\mathrm{router}\;r_{j},\\ 0 & \mathrm{otherwise}. \end{array}\right.$$
- Network connectivity: This is defined as the largest sub-network among *k* formed sub-networks considering the number of mesh nodes (both routers and clients). It can be found as [28]:
  (3) $$\Phi \left(G\right)=max_{i\in \{1,\ldots ,k\}}\left|G_{i}\right|$$
  where $|G_{i}|$, for i∈{1,k}, denotes the size of the $i^{th}$ sub-network, and $G=G_{1}\cup G_{2}\cup \ldots \cup G_{k}$.

**Table 1:** The main notations used in this paper.

Parameter	Description
G=(V,E)	Undirected graph
*V*	Set of mesh nodes
*E*	Set of links between mesh nodes
*M*	Set of mesh routers
*C*	Set of mesh clients
*m*	Number of mesh routers
*n*	Number of mesh clients
$m_{i}$	The *i*-th mesh router
$c_{i}$	The *i*-th mesh client
*R*	Coverage radius of mesh routers
$G_{i}=\left(V_{i},E_{i}\right)$	The *i*-th sub-network
$|G_{i}|$	Size of the *i*-th sub-network
$G_{n}$	Number of sub-networks
ϕ(G)	Network connectivity
ψ(G)	User coverage
$\sigma_{ij}$	Coverage variable
*W*	Width of the dimension
*H*	Height of the dimension
$n_{j}$	Number of covered clients in j-th router
$P_{i}$	Probability of coverage
$P_{j}$	Probability of connectivity
$H_{\mathrm{cov}}$	Coverage entropy
$H_{\mathrm{con}}$	Connectivity entropy

## 3. Maximum Entropy Genetic Algorithm

In this section, we present a detailed explanation of MEGA for mesh router nodes placement. Our methodology consists of two parts: GA and entropy fitness estimation.

### 3.1. Genetic Algorithm

**Figure 2:**
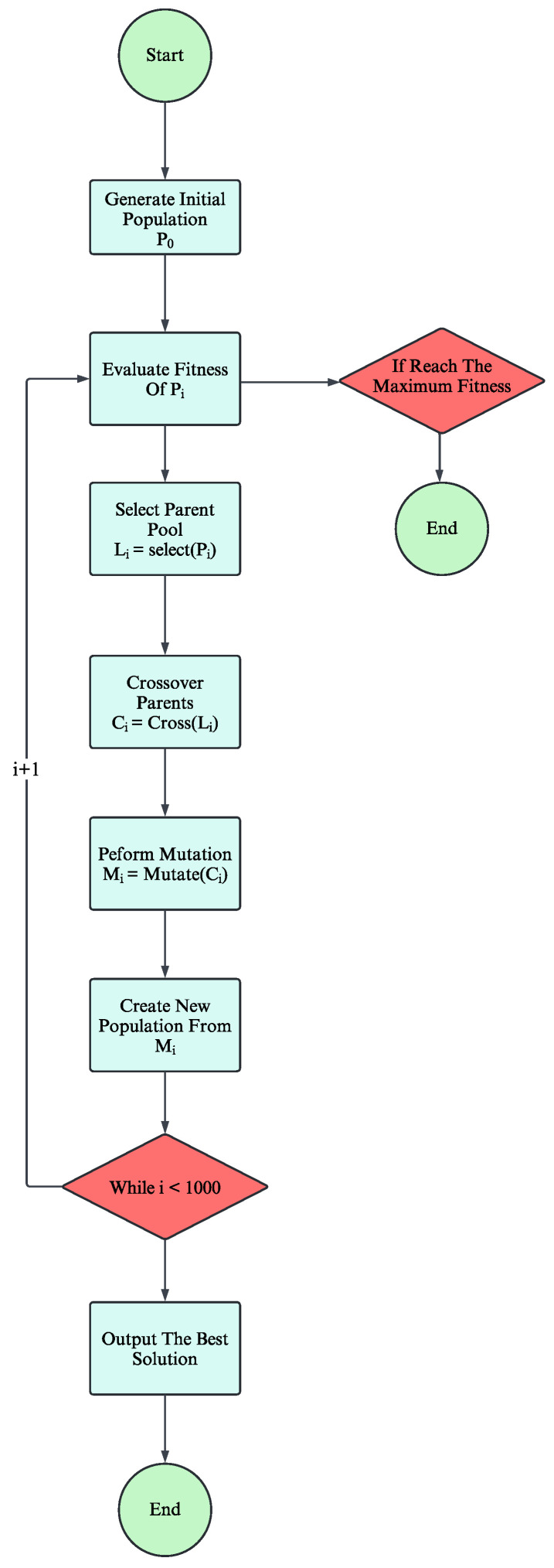
Flowchart of the MEGA.

GAs are adaptive heuristic search algorithms rooted in the principles of natural selection and genetics. This simulation imitates the process of evolution, where individuals, representing potential solutions, compete for resources and opportunities to reproduce [32,33]. Through selection, crossover, and mutation, GA iteratively refines the population, favoring individuals with higher fitness. This emulation of “survival of the fittest” leads to the generation of high-quality solutions for optimization and search problems [34,35]. The flowchart illustrating the MEGA algorithm is presented in Figure 2.

- Initialization: At the beginning, clients are uniformly randomly distributed within a two-dimensional area. Then, to initiate the optimization process, a random set of candidate solutions, specifically mesh routers, is generated. This step involves initializing a population of a specific size, where each individual is represented by a chromosome. The chromosome in our method indicates the positions of mesh routers and consists of two arrays representing the x- and y-coordinates, with its length matching to the number of routers within the clients’ distributed area. After that, the fitness functions of each router within the population are calculated, which will be used for parent selection. A detailed explanation of how fitness is calculated is presented in the next subsection.
- Selection: After evaluating the fitness of the population, we proceed with the selection operator to identify the top-performing individuals for reproduction. We employ a tournament selection strategy, which implies selecting the highest 20 percent of the population based on their fitness values. By sorting the fitness values and selecting the corresponding individuals, we define this subset as parents for the next generation.
- Crossover operators: The crossover operator plays a crucial role in GAs, facilitating the transmission of advantageous genetic traits to future generations and driving evolutionary progress. In our implementation, we use a single-point crossover, where the crossover point is randomly selected within the length of the chromosome. The chromosome is split at this point, with the first part coming from one parent and the second from the other. This diversifies solutions, enhancing the genetic algorithm’s effectiveness in optimizing mesh router nodes placement.
- Mutation operators: Mutation operators in GAs typically lead to minor local changes in the individuals’ chromosome, contrasting with crossover operators. The mutation process introduces variability by randomly altering individual genes within the chromosome. We use a bit-flip mutation approach, where each gene (router position) has a probability of mutation. The mutation rate is adaptive, starting at 10% and decreasing as the algorithm approaches convergence. The likelihood of mutation decreases as fitness approaches its maximum value, leading to optimal solutions.
- Optimal result output: To maintain diversity, we implement an elitist replacement strategy, where the least-fit individuals from the current population are replaced by the offspring generated through crossover and mutation. This ensures that the population continually evolves until it achieves the maximum fitness value, outputting the optimal result.

### 3.2. Entropy Fitness Estimation

In our proposed method, the fitness function is calculated based on Shannon entropy [29]. Entropy is a fundamental concept in information theory that quantifies uncertainty and probability, providing insight into the information content within a system [36,37]. In our algorithm, information includes both the uniform distribution of covered clients and the interconnectivity among mesh routers. These aspects are used for estimating the fitness function by defining connectivity and coverage entropy based on the network topology. The coverage entropy evaluates how covered clients are dispersed by the mesh routers, considering uncertainty in client coverage within the network. Similarly, the connectivity entropy quantifies uncertainty in the interconnections among mesh routers and clients.

**Figure 3:**
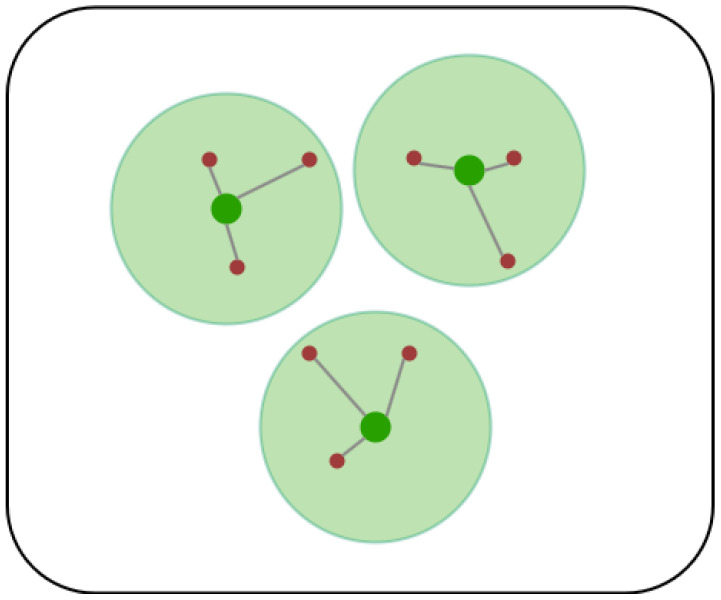
The scenario illustrates an equal coverage probability distribution, where green nodes represent mesh routers with coverage count $n_{j}$ = 3, red nodes represent mesh clients (*n* = 9), and gray lines represent the connectivity between mesh nodes. Each $P_{i}$ is equal to $\frac{1}{3}$ according to Equation (5), resulting in $H_{\mathrm{cov}}$ = 1.

The coverage entropy ($H_{\mathrm{cov}}$) and according probability ($P_{i}$) can be calculated according to the following formula:

(4) $$H_{\mathrm{cov}}=-\frac{\sum_{i}^{m}P_{i}\ln\left(P_{i}\right)}{\ln(m)}$$

(5) $$P_{i}=\frac{n_{j}}{n}$$

where *m* indicates the total number of mesh routers, and $P_{i}$ denotes coverage probability. The entropy calculation includes iterating over each mesh router’s position and verifying the distance to each client. If a client falls within the specified covering radius of the router, it increases the coverage count $n_{j}$. This count is then divided by the total number of clients *n* to determine the coverage probability $P_{i}$. Dividing by ln(m) in the calculation normalizes the entropy value, ensuring that the fitness function of the optimal solution approaches 1. Figure 3 shows an example case where $H_{\mathrm{cov}}$ reaches its maximum value. Each router in the figure covers an equal number of clients, and the number of routers is equal to the number of clients each router covers. This specific configuration ensures that $H_{\mathrm{cov}}$ reaches its maximum value. This means during iteration, each router approaches coverage of the optimal number of clients based on the number of routers.

**Figure 4:**
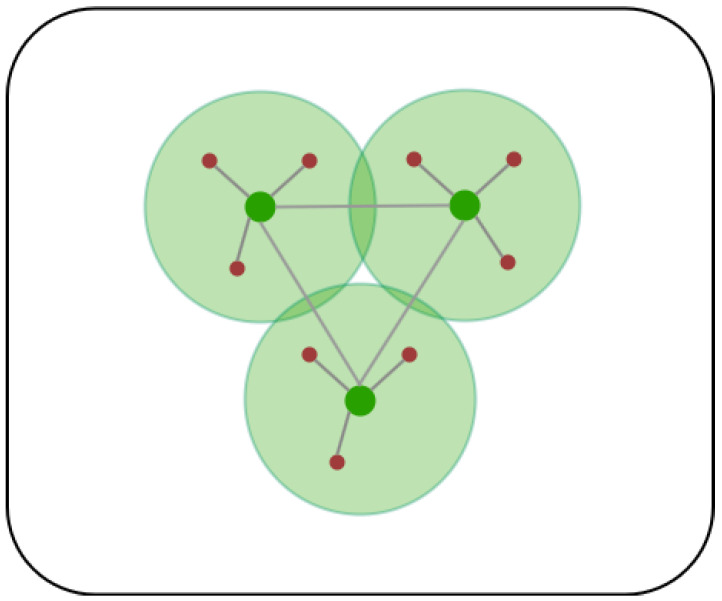
The scenario illustrates a case when $H_{\mathrm{con}}$ = 0, resulting in the best connectivity among nodes according to Equation (6). Here, green nodes represent mesh routers *m*, red nodes represent mesh clients *n*, and gray lines represent the connectivity between mesh nodes.

The connectivity entropy ($H_{\mathrm{con}}$) and according probability ($P_{j}$) can be calculated according to the following formula:

(6) $$\left\{\begin{array}{c} H_{\mathrm{con}}=-\frac{\sum_{j}^{G_{n}}P_{j}\ln\left(P_{j}\right)}{\ln(G_{n})};G_{n}>1\\ H_{\mathrm{con}}=0;G_{n}=1, \end{array}\right.$$

(7) $$P_{j}=\frac{|G_{i}|}{n+m}$$

where $G_{n}$ indicates the number of sub-networks, and $P_{j}$ represents connection probability. We establish connectivity between routers when their Euclidean distance falls within twice the *R*, defining these connected mesh routers and clients within the *R* as components, $|G_{i}|$. We then calculate $P_{j}$ by dividing $|G_{i}|$ by the total count of clients and routers. After that, we calculate $G_{n}$, which represents each cluster of interconnected nodes. It acts as a normalization factor to reduce $H_{\mathrm{con}}$ toward 0. Notably, when the count of $G_{n}$ equals 1, indicating the connection of all components, $H_{\mathrm{con}}$ becomes 0 (Figure 4).

The final fitness function can be calculated according to the following formula:

(8) $$fitness=H_{\mathrm{cov}}-H_{\mathrm{con}}.$$

As $H_{\mathrm{cov}}$ approaches 1 and $H_{\mathrm{con}}$ tends towards 0, the difference between $H_{\mathrm{cov}}$ and $H_{\mathrm{con}}$ balances between these two metrics. The final fitness function converges on 1, reflecting an optimal network configuration.

## 4. Results and Discussion

In this section, we evaluate the performance of the proposed MEGA algorithm for addressing the mesh router nodes placement problem in WMNs. The MEGA algorithm is compared with four top-performing methods: FA [20], GA [15], PSO [10], and COA, as discussed by Mekhmoukh et al. [28].

We assess these algorithms based on three key performance metrics: user coverage, network connectivity, and the value of the objective fitness function. As defined earlier, user coverage is described as the number of mesh clients that are connected to at least one router, while network connectivity is determined by the size of the largest sub-network, measured by the number of mesh nodes across the *k* formed sub-networks. These metrics are used to define the fitness function *f*, which evaluates the quality of potential solutions. The objective function is given as follows [28]:

(9) $$f\left(SC_{c}^{p}\right)=\lambda \cdot \frac{\Psi (G)}{n}+\left(1-\lambda \right)\cdot \frac{\Phi (G)}{m+n}$$

where *G* represents the graph corresponding to the solution $SC_{c}^{p}$, and λ is a floating parameter within the interval [0, 1] which controls the relative importance of the metrics. Therefore, the problem is addressed as a maximization of $f(SC_{c}^{p})$.

MEGA was implemented using Python environment. All tests were conducted using a Core i7 5.2 GHz CPU machine. Simulations were carried out in a rectangular area measuring 2000 m × 2000 m. The number of mesh routers tested varied between 5 and 40, aimed at operating 50 to 300 mesh clients, which were randomly positioned within the test area. Each set of tests included 1000 iterations, and the results were the average outcomes from 50 trials. The simulation parameters are given in Table 2. Our research investigated the impact of different variables, such as the number of mesh clients and routers, as well as the coverage radius.

**Figure 5:**
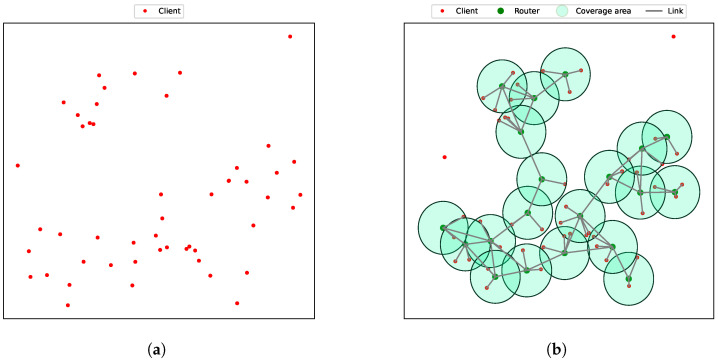
(**a**) The initial random distribution of clients. (**b**) The optimal placement of mesh routers using MEGA, taking into account the distribution of clients. Green nodes denote mesh routers, red nodes indicate mesh clients, and lines between routers show connectivity.

Figure 5 shows an example of a planned WMN using MEGA, with the network designed for a scenario representing 20 mesh routers and 50 clients uniformly distributed over a 4$\;{\mathrm{km}}^{2}$ area.

### 4.1. Impact of Varying the Number of Mesh Clients

**Figure 6:**
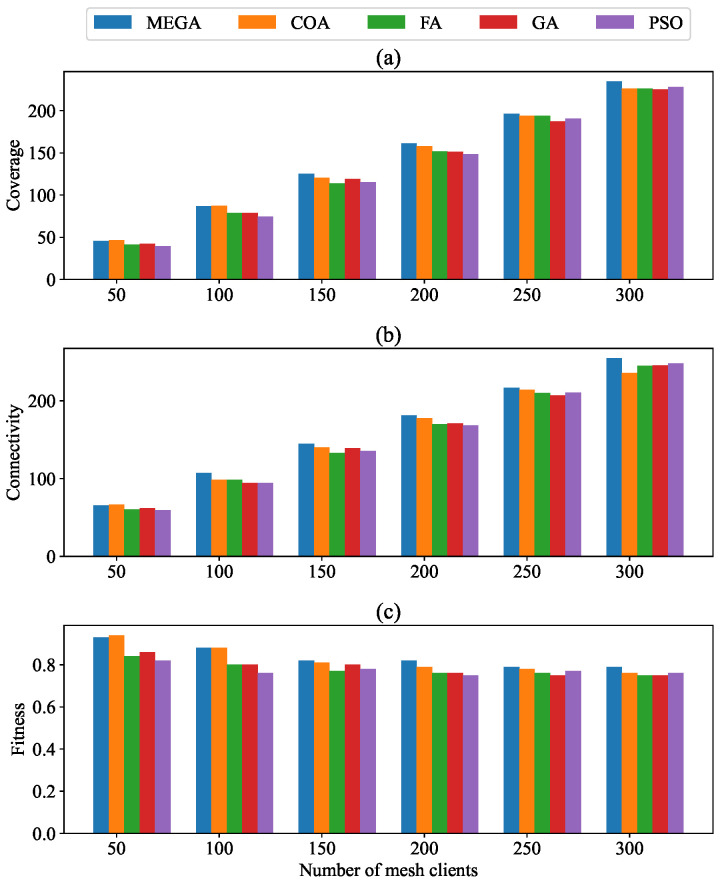
Impact of varying number of mesh clients on (**a**) coverage; (**b**) connectivity; and (**c**) fitness.

In this scenario, we varied the number of mesh clients from 50 to 300 while the number of mesh routers was constant. Table 3 details the influence of increasing the number of mesh clients on user coverage, network connectivity, and the fitness function. In Figure 6, a graphical representation is given. In Table 3, the data for the COA, FA, GA, and PSO algorithms are presented, taken from [28]. Figure 6a illustrates the variation in user coverage as the number of mesh clients increases. We observed a consistent increase in user coverage with an increasing number of clients. Our method demonstrated better performance in client coverage compared to other alternatives: 1.5% more than COA, 7% more than FA, 6.7% more than GA, and 9.2% more than PSO.

Figure 6b illustrates that network connectivity improves as the number of mesh clients increases. It is demonstrated that our method significantly increased network connectivity. More specifically, connectivity was improved by, on average, 3.71%, 6.7%, 6.34%, and 7.3% compared to COA, FA, GA, and PSO, respectively. The results shown in Figure 6c illustrate a decline in fitness values as the number of mesh clients increases, necessitating more routers to keep coverage. With a fixed number of mesh routers, newly added clients might not be covered, resulting in reduced coverage and connectivity, which impacts the fitness value. The obtained results revealed that MEGA performed better than COA, FA, GA, and PSO.

### 4.2. Impact of Varying the Number of Mesh Routers

**Figure 7:**
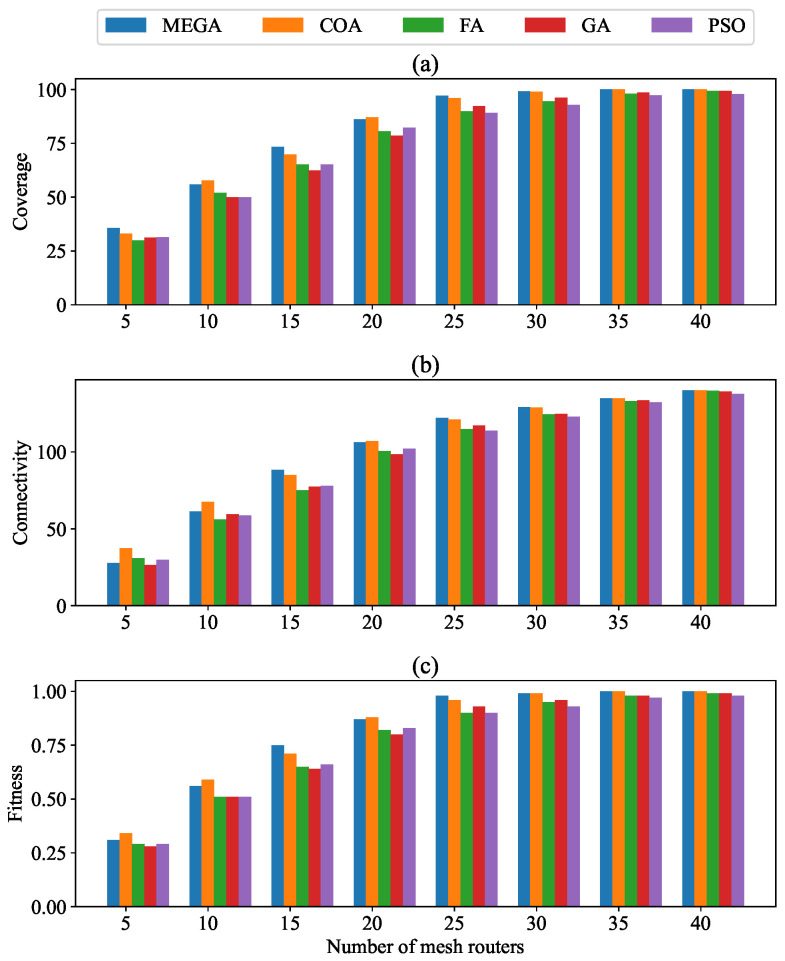
Impact of varying number of mesh routers on: (**a**) coverage; (**b**) connectivity; and (**c**) fitness.

The influence of varying the number of mesh routers (from 5 to 40) on coverage, connectivity, and overall fitness value are given in Table 4 and Figure 7. In Table 4, the data for the COA, FA, GA, and PSO algorithms are displayed, taken from [28]. As depicted in Figure 7a, the coverage of users improved with an increase in mesh routers. More specifically, the coverage under MEGA was improved on average by 1.7%, 9.9%, 10.2%, and 9.8% in comparison to the COA, FA, GA, and PSO algorithms, respectively. Figure 7b illustrates that network connectivity also rises with an increase in mesh routers. This increase results from the reduction in the number of isolated subnetworks as additional routers help to eliminate gaps, forming larger sub-networks. Finally, this leads to the formation of a single, extensive subnetwork encompassing all mesh nodes. The MEGA algorithm achieved the largest subnetwork compared to others, with average connectivity improvements of 5.4%, 6.2%, and 4.5% over FA, GA, and PSO, excluding COA, which performed 5.2% better.

Figure 7c demonstrates that the fitness value correlates positively with the number of mesh routers. As the number of routers increases, the fitness value improved across all algorithms. The proposed MEGA consistently surpassed COA, FA, GA, and PSO in performance when the number of mesh routers exceeded 10. This trend suggests that increasing router density not only enhances coverage and connectivity, but also significantly enhances overall network performance.

### 4.3. Impact of Varying the Router Coverage Radius

**Figure 8:**
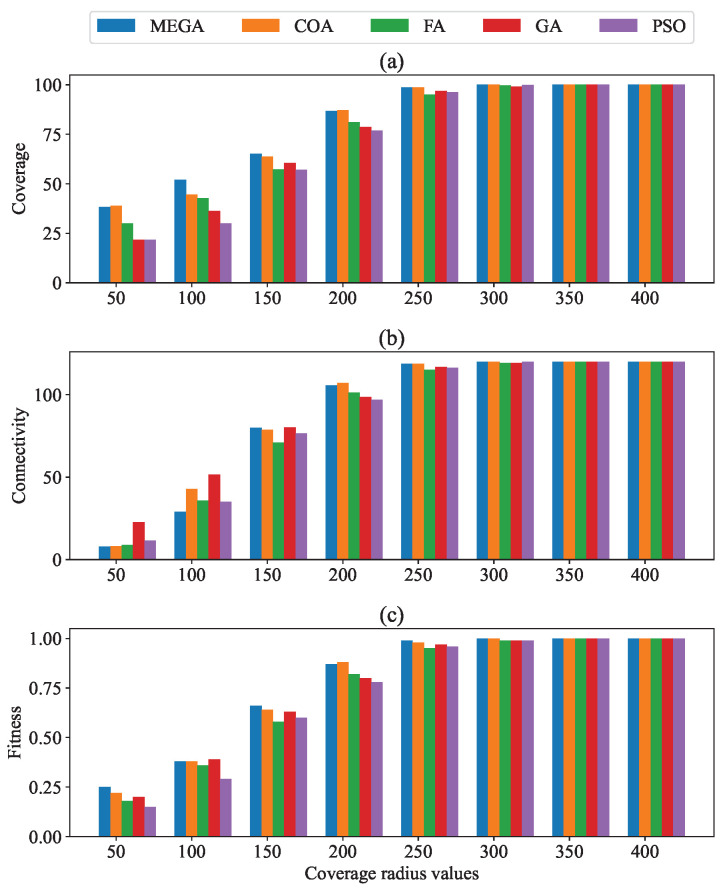
Impact of varying coverage radius values on (**a**) coverage; (**b**) connectivity; and (**c**) fitness.

Table 5 and Figure 8 illustrate the influence of varying the coverage radius of mesh routers from 50 to 400 m on coverage, connectivity, and fitness values. In Table 5, the data for the COA, FA, GA, and PSO algorithms are shown, taken from [28]. Figure 8a shows the impact of expanding the coverage radius on network coverage. The results indicate that as each mesh router’s coverage radius was extended, there was a corresponding increase in the coverage metric. Specifically, when the coverage radius exceeded 300 m, most routers were able to cover almost all mesh clients. The MEGA exceeded the performance of other algorithms across all scenarios, improving client coverage on average by 2.8%, 12.5%, 23.4%, and 30% compared to COA, FA, GA, and PSO, respectively.

Figure 8b investigates how network connectivity is influenced by the router’s coverage radius. As the coverage radius of each router increases, the connectivity of the network also increases. This enhancement occurs because each router can cover more clients and establish connections with other routers, thus expanding the largest subnetwork.

Figure 8c indicates that the fitness value improves as the mesh router coverage radius increases. It is demonstrated that the MEGA surpassed other algorithms in enhancing fitness values across different coverage radius settings. More precisely, the fitness value was improved on average by 2.7%, 11.6%, 6.5%, and 20% over COA, FA, GA, and PSO, respectively.

## 5. Conclusions

In this work, we introduced MEGA, a novel Maximum Entropy Genetic Algorithm designed to tackle the complex challenge of mesh router node placement in WMNs. Efficient node placement is vital in WMNs, as it directly impacts network performance, coverage, and connectivity. The method of calculating the fitness function through entropy ensures a uniformly distributed placement of mesh router nodes while considering the positions of mesh clients. By focusing on both network connectivity and user coverage, MEGA provides a robust solution to this NP-hard problem. We considered scenarios in which mesh clients are stationary, with known positions a priori, and developed an optimization model aimed at maximizing these two objectives.

To validate MEGA, we rigorously tested it across various scenarios, adjusting key parameters such as the number of mesh clients, routers, and coverage radius. Our simulation results demonstrate that MEGA outperforms existing algorithms, including COA, FA, GA, and PSO, particularly in achieving higher network connectivity and user coverage. Among these, the COA-based method ranks second, leveraging COA’s recent meta-heuristic approach, which employs few tuning parameters and exhibits strong capabilities in exploration and exploitation. Following COA, the FA method, known as the Firefly Optimization Algorithm, effectively mimics firefly behavior to balance local and global search strategies, making it well-suited for complex optimization tasks.

These findings underscore the effectiveness of entropy-based approaches in addressing intricate node placement challenges in WMNs. In future work, we aim to improve MEGA by optimizing its performance across various network topologies and complex environments, including 3D spaces and scenarios with obstacles or interference. 

## Figures and Tables

**Table 2:** Parameter values considered in our simulations.

Parameter	Value	Default Value
*n*	[50, 300]	100
*m*	[5, 40]	20
*R*	[50, 400] m	200 m
*W*	2000 m	2000 m
*H*	2000 m	2000 m
Population size	50	50
Number of runs	50	50
Number of iterations	1000	1000

**Table 3:** Coverage, connectivity, and fitness under various numbers of mesh clients.

n	50	100	150	200	250	300
**Coverage**						
MEGA	45.7	87	**125**	**161.4**	**196.5**	**234.6**
COA	**46.74**	**87.12**	120.32	157.88	194	226
FA	41.44	78.74	113.82	151.63	194	222.16
GA	42.26	78.6	118.86	151.1	187.13	225.33
PSO	39.63	74.56	115.33	148.53	190.66	228.03
**Connectivity**						
MEGA	65.7	107	**145**	**181.4**	**216.5**	**254.6**
COA	**66.5**	**107.12**	140.32	177.5	214	248.2
FA	60.38	98.24	132.8	170.1	210.16	244.84
GA	62	98.6	138.86	171.1	207.13	245.33
PSO	59.63	94.4	135.33	168.53	210.66	248.03
**Fitness**						
MEGA	0.93	**0.88**	**0.82**	**0.82**	**0.79**	**0.79**
COA	**0.94**	0.88	0.81	0.79	0.78	0.76
FA	0.84	0.80	0.77	0.76	0.75	0.75
GA	0.86	0.80	0.80	0.76	0.76	0.75
PSO	0.82	0.76	0.78	0.75	0.77	0.76

**Table 4:** Coverage, connectivity, and fitness under various numbers of mesh routers.

m	5	10	15	20	25	30	35	40
**Coverage**								
MEGA	**35.6**	55.9	**73.3**	86.2	**97.2**	**99.1**	**100**	**100**
COA	32.96	**57.7**	69.9	**87**	96	99	100	100
FA	29.86	52	65.2	80.6	89.8	94.6	98.06	99.36
GA	31.23	49.93	62.43	78.6	92.23	96.26	98.53	99.36
PSO	31.33	49.86	65.2	82.33	89.06	92.86	97.33	97.86
**Connectivity**								
MEGA	27.7	61.2	**88.3**	106.2	**122.2**	**129.1**	**135**	**140**
COA	**37.42**	**67.43**	84.9	**107**	121	129	135	140
FA	30.86	56	75	100.6	114.8	124.6	133	139.76
GA	26.46	59.43	77.43	98.6	117.23	124.8	133.53	139.36
PSO	29.86	58.58	77.96	102.2	113.8	122.86	132.33	137.86
**Fitness**								
MEGA	0.31	0.56	**0.75**	**0.88**	**0.98**	**0.99**	**1**	**1**
COA	**0.34**	**0.59**	0.71	0.88	0.96	0.99	1	1
FA	0.29	0.51	0.65	0.82	0.9	0.95	0.98	0.99
GA	0.28	0.51	0.64	0.8	0.93	0.96	0.98	0.99
PSO	0.29	0.51	0.66	0.83	0.9	0.93	0.97	0.98

**Table 5:** Coverage, connectivity, and fitness under various coverage radius values.

CR	50	100	150	200	250	300	350	400
**Coverage**								
MEGA	38.3	**52.1**	**65.2**	86.8	98.6	**100**	**100**	**100**
COA	**38.88**	44.6	63.78	**87.12**	**98.72**	100	100	100
FA	29.9	42.76	57.2	81.2	95.13	99.66	100	100
GA	21.73	36.36	60.53	78.6	96.76	99.16	100	100
PSO	21.63	29.9	57.1	76.93	96.26	99.86	100	100
**Connectivity**								
MEGA	7.9	29	**80**	105.5	118.6	**120**	**120**	**120**
COA	**8.26**	**42.88**	78.72	**107.12**	**118.72**	120	120	120
FA	9	35.73	70.82	101.2	115.13	119.2	120	120
GA	22.63	51.63	80.1	98.6	116.76	119.16	120	120
PSO	11.63	34.96	76.63	96.93	116.26	119.86	120	120
**Fitness**								
MEGA	**0.25**	0.38	**0.66**	0.87	**0.99**	**1**	**1**	**1**
COA	0.22	0.38	0.64	**0.88**	0.98	1	1	1
FA	0.18	0.36	0.58	0.82	0.95	0.99	1	1
GA	0.2	**0.39**	0.63	0.8	0.97	0.99	1	1
PSO	0.15	0.29	0.6	0.78	0.96	0.99	1	1

## Data Availability

Data are contained within the article.

## References

[1] Barolli A., Bylykbashi K., Qafzezi E., Sakamoto S., Barolli L. (2023). A comparison study of Weibull, normal and Boulevard distributions for wireless mesh networks considering different router replacement methods by a hybrid intelligent simulation system. J. Ambient Intell. Humaniz. Comput..

[2] Hussain M.I., Ahmed N., Ahmed M.Z.I., Sarma N. (2022). QoS provisioning in wireless mesh networks: A survey. Wirel. Pers. Commun..

[3] Nouri N.A., Aliouat Z., Naouri A., Hassak S.A. (2023). Accelerated PSO algorithm applied to clients coverage and routers connectivity in wireless mesh networks. J. Ambient Intell. Humaniz. Comput..

[4] Janjua M.B., Duranay A.E., Arslan H. (2020). Role of Wireless Communication in Healthcare System to Cater Disaster Situations Under 6G Vision. Front. Commun. Netw..

[5] Rethfeldt M., Brockmann T., Beichler B., Haubelt C., Timmermann D. (2021). Adaptive Multi-Channel Clustering in IEEE 802.11s Wireless Mesh Networks. Sensors.

[6] Taleb S.M., Meraihi Y., Gabis A.B., Mirjalili S., Ramdane-Cherif A. (2022). Nodes placement in wireless mesh networks using optimization approaches: A survey. Neural Comput. Appl..

[7] Seetha S., Anand John Francis S., Grace Mary Kanaga E. (2021). Optimal placement techniques of mesh router nodes in wireless mesh networks. Proceedings of the 2nd EAI International Conference on Big Data Innovation for Sustainable Cognitive Computing: BDCC 2019.

[8] Amaldi E., Capone A., Cesana M., Filippini I., Malucelli F. (2008). Optimization models and methods for planning wireless mesh networks. Comput. Netw..

[9] Qiu L., Bahl P., Rao A., Zhou L. (2006). Troubleshooting wireless mesh networks. ACM SIGCOMM Comput. Commun. Rev..

[10] Lin C.C. (2013). Dynamic router node placement in wireless mesh networks: A PSO approach with constriction coefficient and its convergence analysis. Inf. Sci..

[11] Lin C.C., Li Y.S., Deng D.J. (2014). A bat-inspired algorithm for router node placement with weighted clients in wireless mesh networks. Proceedings of the 9th International Conference on Communications and Networking in China.

[12] Lin C.C., Tseng P.T., Wu T.Y., Deng D.J. (2016). Social-aware dynamic router node placement in wireless mesh networks. Wirel. Netw..

[13] Binh L.H., Truong T.K. (2022). An Efficient Method for Solving Router Placement Problem in Wireless Mesh Networks Using Multi-Verse Optimizer Algorithm. Sensors.

[14] Sayad L., Bouallouche-Medjkoune L., Aissani D. (2018). A simulated annealing algorithm for the placement of dynamic mesh routers in a wireless mesh network with mobile clients. Internet Technol. Lett..

[15] Oda T., Elmazi D., Barolli A., Sakamoto S., Barolli L., Xhafa F. (2016). A genetic algorithm-based system for wireless mesh networks: Analysis of system data considering different routing protocols and architectures. Soft Comput..

[16] Xhafa F., Barolli A., Sánchez C., Barolli L. (2011). A simulated annealing algorithm for router nodes placement problem in wireless mesh networks. Simul. Model. Pract. Theory.

[17] Xhafa F., Sánchez C., Barolli L. (2012). Local search methods for efficient router nodes placement in wireless mesh networks. J. Intell. Manuf..

[18] Xhafa F., Sánchez C., Barolli A., Takizawa M. (2015). Solving mesh router nodes placement problem in wireless mesh networks by tabu search algorithm. J. Comput. Syst. Sci..

[19] Sayad L., Bouallouche-Medjkoune L., Aissani D. (2020). A chemical reaction algorithm to solve the router node placement in wireless mesh networks. Mob. Netw. Appl..

[20] Sayad L., Aissani D., Bouallouche-Medjkoune L. (2018). Placement optimization of wireless mesh routers using firefly optimization algorithm. Proceedings of the 2018 International Conference on Smart Communications in Network Technologies (SaCoNeT).

[21] Xhafa F., Sanchez C., Barolli L., Spaho E. (2010). Evaluation of genetic algorithms for mesh router nodes placement in wireless mesh networks. J. Ambient Intell. Humaniz. Comput..

[22] Tang L., Wang Z., Huang J., Bian L. (2019). A general purpose deployment method for wireless mesh network. Int. J. Internet Protoc. Technol..

[23] De Marco G. MOGAMESH: A multi-objective algorithm for node placement in wireless mesh networks based on genetic algorithms. Proceedings of the 2009 6th International Symposium on Wireless Communication Systems.

[24] Bello O.M., Taiwe K.D. Mesh node placement in wireless mesh network based on multiobjective evolutionary metaheuristic. Proceedings of the International Conference on Internet of things and Cloud Computing.

[25] Farahani R.Z., SteadieSeifi M., Asgari N. (2010). Multiple criteria facility location problems: A survey. Appl. Math. Model..

[26] Zavala G.R., Nebro A.J., Luna F., Coello Coello C.A. (2014). A survey of multi-objective metaheuristics applied to structural optimization. Struct. Multidiscip. Optim..

[27] Alothaimeen I., Arditi D. (2019). Overview of multi-objective optimization approaches in construction project management. Multicriteria Optimization-Pareto-Optimality and Threshold-Optimality.

[28] Taleb S.M., Meraihi Y., Gabis A.B., Mirjalili S., Zaguia A., Ramdane-Cherif A. (2022). Solving the mesh router nodes placement in wireless mesh networks using coyote optimization algorithm. IEEE Access.

[29] Shannon C.E. (1948). A mathematical theory of communication. Bell Syst. Tech. J..

[30] Oda T., Liu Y., Sakamoto S., Elmazi D., Barolli L., Xhafa F. (2015). Analysis of mesh router placement in wireless mesh networks using Friedman test considering different meta-heuristics. Int. J. Commun. Netw. Distrib. Syst..

[31] Benyamina D., Hafid A., Gendreau M. (2011). Wireless mesh networks design—A survey. IEEE Commun. Surv. Tutor..

[32] Mitchell M. (1998). An Introduction to Genetic Algorithms.

[33] Goldberg D.E. (1994). Genetic and evolutionary algorithms come of age. Commun. ACM.

[34] Holland J.H. (1992). Adaptation in Natural and Artificial Systems: An Introductory Analysis with Applications to Biology, Control, and Artificial Intelligence.

[35] Eiben A.E., Smith J.E. (2015). Introduction to Evolutionary Computing.

[36] Csiszár I., Körner J. (2011). Information Theory: Coding Theorems for Discrete Memoryless Systems.

[37] Cover T.M. (1999). Elements of Information Theory.

